# Skeletal muscle as a regulator of the longevity protein, Klotho

**DOI:** 10.3389/fphys.2014.00189

**Published:** 2014-06-17

**Authors:** Keith G. Avin, Paul M. Coen, Wan Huang, Donna B. Stolz, Gwendolyn A. Sowa, John J. Dubé, Bret H. Goodpaster, Robert M. O'Doherty, Fabrisia Ambrosio

**Affiliations:** ^1^Department of Physical Medicine and Rehabilitation, University of PittsburghPittsburgh, PA, USA; ^2^Division of Geriatric Medicine, Department of Medicine, University of PittsburghPA, USA; ^3^Division of Endocrinology and Metabolism, Department of Medicine, University of PittsburghPittsburgh, PA, USA; ^4^Department of Health and Physical Education, University of PittsburghPittsburgh, PA, USA; ^5^Department of Cell Biology and Physiology, University of PittsburghPittsburgh, PA, USA; ^6^McGowan Institute for Regenerative Medicine, University of PittsburghPittsburgh, PA, USA

**Keywords:** skeletal muscle, Klotho, aging, regeneration, exercise

## Abstract

Klotho is a powerful longevity protein that has been linked to the prevention of muscle atrophy, osteopenia, and cardiovascular disease. Similar anti-aging effects have also been ascribed to exercise and physical activity. While an association between muscle function and Klotho expression has been previously suggested from longitudinal cohort studies, a direct relationship between circulating Klotho and skeletal muscle has not been investigated. In this paper, we present a review of the literature and preliminary evidence that, together, suggests Klotho expression may be modulated by skeletal muscle activity. Our pilot clinical findings performed in young and aged individuals suggest that circulating Klotho levels are upregulated in response to an acute exercise bout, but that the response may be dependent on fitness level. A similar upregulation of circulating Klotho is also observed in response to an acute exercise in young and old mice, suggesting that this may be a good model for mechanistically probing the role of physical activity on Klotho expression. Finally, we highlight overlapping signaling pathways that are modulated by both Klotho and skeletal muscle and propose potential mechanisms for cross-talk between the two. It is hoped that this review will stimulate further consideration of the relationship between skeletal muscle activity and Klotho expression, potentially leading to important insights into the well-documented systemic anti-aging effects of exercise.

## Introduction

Mankind has long sought means to extend longevity and counteract the effects of aging on physical functioning. Modern day scientific discoveries have made considerable strides in our biological understanding of contributing factors in the aging process. Such discoveries are critical for the development of therapeutic strategies to prevent, delay or reverse age-related declines.

Skeletal muscle is the largest organ of the human body, and it comprises over 40% of the body's mass in non-obese individuals. It is therefore not surprising that alterations to the skeletal muscle contractile unit have profound effects on overall organismal health. Age-related declines are manifest by a decreased ability for aged skeletal muscle to respond to physiological stimuli such as muscle loading or acute injury. Indeed, older adults often exhibit an age-related reduction in the number and size of muscle fibers, known as sarcopenia (Lexell, 1995). The subsequent sarcopenia-related muscle weakening and atrophy soon leads to the onset of decreased mobility. Secondary effects of sarcopenia include an increased risk for falls (Tinetti, 1987), declines in physical functioning (Rantanen et al., 1997) and a decreased participation in activities of daily living (Tinetti, 1987; Szulc et al., 2005; Reid et al., 2008). Macro-level deficits are, at least partially, the result of age-related molecular and cellular alterations. These alterations include impaired metabolic pathways (i.e., insulin/IGF, forkhead transcription factor, and mTOR signaling) and altered muscle maintenance systems (i.e., ubiquitin-proteasome system, autophagy-lysosome system) (Sandri et al., 2013). In addition, muscle stem (satellite) cells (MuSCs), responsible for dictating skeletal muscle regenerative capacity, display impaired functioning with increasing age (reviewed in Conboy and Rando, 2012). With age, these cells demonstrate a decline in regenerative potential owing to altered cellular proliferation and myogenic differentiation (reviewed in Mann et al., 2011; Conboy and Rando, 2012). As the number of elderly individuals in the United States grows, functional detriments resulting from these age-related declines in skeletal muscle maintenance and healing capacity will increasingly represent an important public health burden. The development of methods to promote healthy aging has, therefore, become more important than ever.

There is abundant evidence that exercise may be effective in preventing, delaying or reversing the effect of age on tissue health and functioning. The anti-aging physiological benefits of physical activity/exercise have been well documented and include the prevention of muscle wasting, cardiovascular diseases, and hypertension (see Table 1). Accordingly, physical fitness level is an important predictor of both being able to live independently into old age and all-cause mortality (Myers et al., 2002; Gulati et al., 2003; Myers, 2003). Studies have suggested that improving physical fitness may decrease the risk of death by up to 44% (Blair et al., 1995). Inherent to physical activity or exercise is skeletal muscle contractile activity. While other benefits of exercise, such as improved cardiovascular health, may not be discounted, adequate skeletal muscle functioning is likely a *sine qua non* in the anti-aging effects of exercise (Castillo-Garzon et al., 2006). Indeed, handgrip strength, a surrogate measure for total body muscle strength (Rantanen et al., 2003), has been shown in previous studies to be a reliable marker of wellbeing (Lord et al., 2003; Chang et al., 2004; Hulsmann et al., 2004) and is a potent predictor of both the expectancy to live independently and mortality (Castillo-Garzon et al., 2006). Perhaps even more compelling are findings that grip strength in healthy, middle aged men is predictive of functional limitations and disability 25 years later (Rantanen et al., 1999). Based on this and other studies, Rantanen and colleagues suggested that muscle strength may serve as a marker of “physiological reserve” even in midlife, and that the extent of this reserve may be indicative of vulnerability to disease and disability into old age (Rantanen et al., 2012).

**Table 1:** **Summary depicting overlapping physiological and functional responses to Klotho expression and exercise/physical activity**.

**Age-related phenotype**	**Klotho**	**Exercise**
Gait function	Positive association: gait dysfunction observed in *Kl* mice; in humans, higher klotho levels are associated with improved scores on tests of lower extremity strength and functioning (Kuro-o et al., 1997; Crasto et al., 2012)	Positive association: improved fitness is associated with greater walking capacity (Peeters and Mets, 1996; Newman et al., 2006; Simonsick et al., 2006)
Cardiovascular disease (CVD)	Negative association: *Kl* mice demonstrate arteriosclerosis (Kuro-o et al., 1997). In humans, higher klotho levels were associated with lower likelihood of CVD (Semba et al., 2011)	Negative association: sedentary behavior is positively associated with mortality and CVD (Matthews et al., 2008; Warren et al., 2010; Chomistek et al., 2013)
Osteoporosis	Negative association: decreased Klotho levels are associated with decreased bone mineral density of hindlimb bones, primarily as a result of decreased cortical bone thickness (Kuro-o et al., 1997)	Negative association: lower physical activity is associated with decreased bone mineral density (Martyn-St James and Carroll, 2009; Hamilton et al., 2010)
Cognitive function	Positive association: *Kl* mice display impaired novel-object recognition and associative memory (Nagai et al., 2003; Kuang et al., 2014). Klotho levels have been shown to be lower in those with Alzheimer's disease (Semba et al., 2014)	Positive association: exercise promotes brain plasticity and has been shown to halt, delay and/or reverse the effects of aging on cognitive function (reviewed in Foster et al., 2011)
Angiogenesis	Positive association: *Kl* mice demonstrate significantly decreased skeletal muscle capillary density and evidence of angiogenesis following an ischemic injury (Fukino et al., 2002; Shimada et al., 2004)	Positive association: muscle loading results in a significantly increased skeletal muscle capillary density and the promotion of angiogenesis (Hoier et al., 2012)
Resistance to stress	Positive association: Klotho protects cells from apoptosis induced by oxidative stress (Yamamoto et al., 2005)	Positive association: whereas acute stress may induce oxidative stress and reactive oxygen species accumulation, chronic training has been shown to promote upregulation of antioxidants (Gomez-Cabrera et al., 2008; Mangner et al., 2013)
Tissue regeneration	Positive association: *Kl* mice display increase Wnt signaling, decreased stem cell numbers and an impaired regenerative response (Liu et al., 2007)	Positive association: muscle loading enhances the participation of stem cells in muscle regeneration (Ambrosio et al., 2010; Distefano et al., 2013). An acute bout of exercise results in increased satellite cell number (Dreyer et al., 2006; Kosek et al., 2006; Cermak et al., 2012), while chronic, repeated bouts mRNA expression of myogenic markers and reduces mRNA expression for inhibitors of myogenesis (Carey, 2007; Costa et al., 2007). Endurance exercise increases satellite cell content by 30–58% (Charifi et al., 2003; Verney et al., 2008)

What is the biological basis underlying the relationship between skeletal muscle contractile activity and tissue declines? The systemic anti-aging benefits of exercise suggest that humoral factors may play a role. Indeed, a potential relationship between the circulating longevity protein, Klotho, and the pathogenesis of sarcopenia was recently suggested by a large, population-based study of aging by Semba et al. (2011). Klotho has been called an “aging suppressor gene” and has been suggested to delay age-related declines in physiological functioning (Kuro-o et al., 1997). The protein product of this gene has been detected in the circulatory system of both animals and humans (Kuro-o et al., 1997; Xiao et al., 2004), although its serum concentration gradually declines with increasing age (Xiao et al., 2004). In two studies, a strong association between plasma Klotho expression and skeletal muscle strength (Semba et al., 2012) and functioning (Crasto et al., 2012) were revealed. As summarized in Table 1, there are many parallels between age-related processes/pathologies that are regulated by Klotho and those regulated by exercise/or physical activity. Given these parallels, we were driven by a curiosity as to whether there may be a cross-talk between skeletal muscle activity and expression of this longevity protein. The purpose of this “Hypothesis and Theory” style manuscript is to present a literature review and preliminary results from our laboratory in support of our novel hypothesis that biochemical events originating within skeletal muscle are important triggers of Klotho expression. Specifically, we hypothesize that skeletal muscle contractile activity modulates circulating Klotho expression and that this regulation may play a role in the well-documented systemic anti-aging effects of exercise. Moreover, we propose that Klotho may participate in the skeletal muscle regenerative cascade and that age-related declines in Klotho levels may contribute to the decreased ability of aged skeletal muscle to heal after injury.

## The regulation and roles of klotho

Klotho is present in both membrane-bound and secreted forms. The secreted form is generated through alternative splicing or through shedding of the extracellular domain of the transmembrane protein by membrane-anchored proteases (Kuro-o et al., 1997), including A disintegrin and metalloproteinase domain–containing protein 10 (ADAM10), ADAM metallopeptidase domain 17 (ADAM17) and Beta-secretase 1 (BACE 1) (Chen et al., 2007). The secreted form of Klotho has been shown to exert biological effects throughout the body, indicating its potential as a humoral factor. The membrane-bound version serves as an obligate co-receptor for fibroblast growth factor-23 (FGF23) signaling (Kurosu et al., 2006), whereas the secreted protein functions independently of FGF23. Since its original discovery in 1997, two homologs of Klotho, α and β, have been identified, with β-Klotho sharing 41% amino acid identity with α-Klotho. Throughout this paper, the term “Klotho” refers to the α-Klotho homolog.

**Figure 1:**
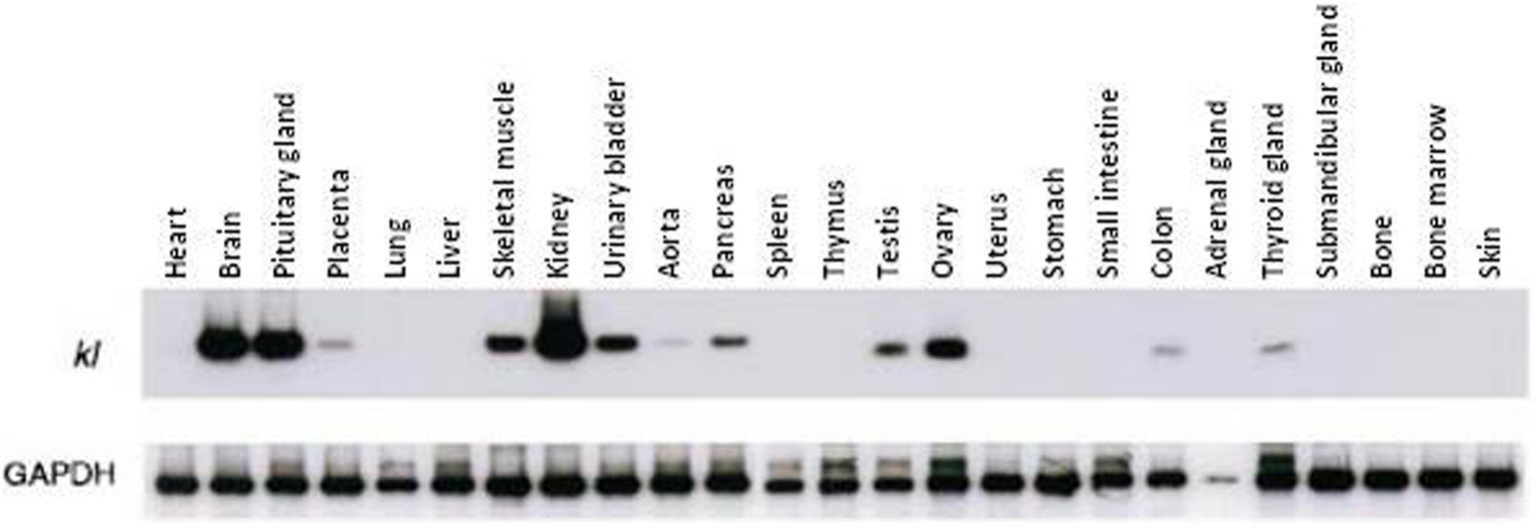
**Klotho gene expression in multiple tissues.** Kuro-o and colleagues quantified Klotho gene expression of multiple tissues using RT–PCR. The kidney, brain and pituitary gland demonstrate the greatest expression, whereas skeletal muscle, ovaries and testes express Klotho to a relatively lesser extent. (Reproduced from Kuro-o et al., 1997).

Klotho is most highly expressed in the kidney, brain and pituitary gland, and is present in lower levels within skeletal muscle, the urinary bladder, the ovary and the testes (Kuro-o et al., 1997). Trace amounts of Klotho are also observed in the placenta, aorta, colon, and the thyroid gland (Kuro-o et al., 1997) (See Figure 1, reprinted from Kuro-o et al., 1997). β-Klotho is expressed in adipose tissue, as well as the liver and pancreas (Ito et al., 2000).

The intriguing role for Klotho in age-related declines was first identified through the serendipitous discovery of the *Klotho* knockout mouse (*kl* mouse). *Kl* mice display limited membrane-bound and secreted Klotho protein expression as a result of an ectopic DNA insertion into the Klotho gene. This mutation results in lifespans approximately 5–6% that of their wild type counterparts (10–12 weeks and 2.5–3 years, respectively) (Kuro-o et al., 1997). Although the exact cause of death is unknown, within their short lifespan, the decreased longevity of *kl* mice is consistent with a myriad of aging phenotypes, including decreased activity levels, hypokinesis, gait disturbance, atherosclerosis, cognitive impairment, sarcopenia and an impaired wound repair process. Importantly, genetic up-regulation of Klotho in mice reverses age-related declines in the physiological functioning of various tissues and extends lifespan by 20–30% beyond the normal lifespan of wild type mice (Kurosu et al., 2005).

## The effect of exercise and training on plasma klotho expression

There is limited data exploring the relationship between parameters of physical health/fitness and Klotho. Analyses obtained from the Invecchiare in Chianti “Aging in the Chianti Area” (InCHIANTI) study, a population-based longitudinal study, revealed that low plasma levels of Klotho are associated with decreased activities of daily living in older individuals (Crasto et al., 2012). Low plasma Klotho levels have also been associated with a lower score on the Short Physical Performance Battery (a test of lower extremity strength and functioning) (Crasto et al., 2012), as well as poor muscle strength in older, community dwelling adults (Semba et al., 2012). Like poor handgrip strength (Rantanen et al., 1999), low plasma Klotho levels were also shown to be an independent predictor of all-cause mortality (Semba et al., 2011). While reports failed to observe any direct relationship between “physical activity” and circulating Klotho levels, in these studies, physical activity was a self-reported measure of behavior [ranked on a progression scale from 0 (inactive) to 7 (intense exercise several times/week)] and may not necessarily be indicative of physical fitness levels, *per se*.

Clinical correlations between Klotho expression and skeletal muscle strength are consistent with a pre-clinical study where grip strength and running endurance were compared among *kl* mice, Klotho overexpressing mice (*EFmKL46*) and wild-type (WT) control mice (Phelps et al., 2013). *Kl* mice were significantly weaker, and displayed strength of ~50% less than both *EFmKL46* and WT-controls (there was no difference between *EFmKL46* and WT-controls). Interestingly, *kl* mice ran on a running wheel at the same speed as the other two groups, but they demonstrated an endurance capacity ~60% less than that of *EFmKL46* and WT-controls.

**Figure 2:**
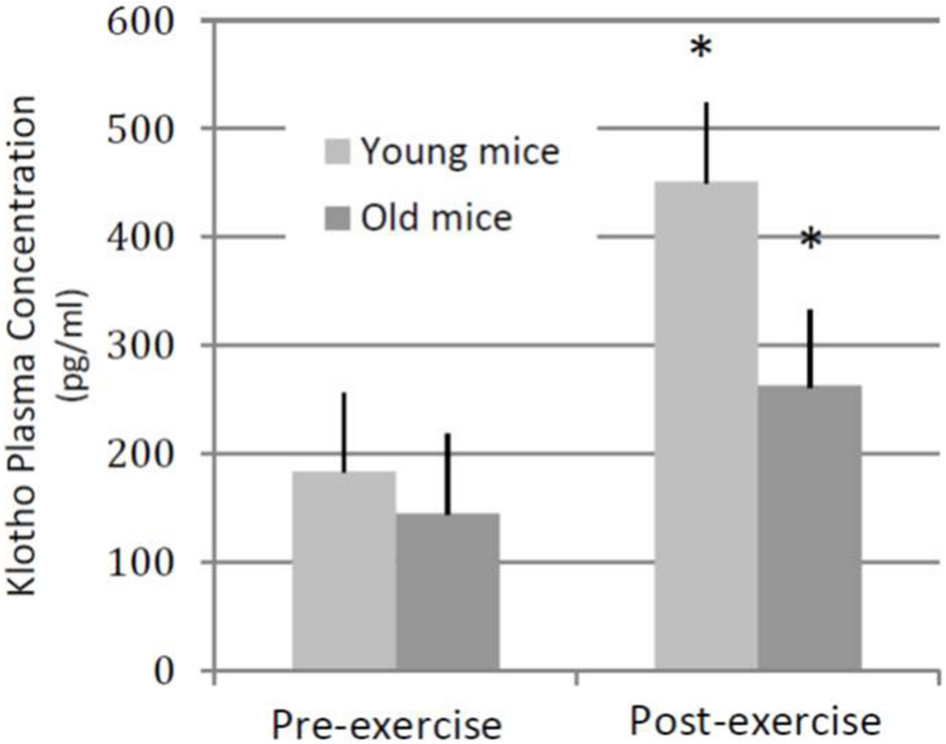
**Plasma Klotho levels following an acute bout of exercise in young and old mice.** Young (3–4 months old, *n* = 4) and aged (22–24 months old, *n* = 4) wild-type mice performed a single session of 45-min, 0° incline treadmill running. In both groups of animals, plasma Klotho levels increased significantly following an exercise bout. ^*^denotes significantly different from pre-training values (within age group).

As a first step to probe the relationship between physical activity, age and Klotho expression, we quantified the effect of an acute exercise bout on circulating Klotho levels in both young (3–4 months) and aged (22–24 months) C57Bl6/J mice. The acute exercise consisted of 45-min of treadmill running at a 0° incline, performed at approximately 70% maximal aerobic capacity (VO_2_max) (Schefer and Talan, 1996). Immediately after exercise, we observed a significant increase in circulating Klotho levels in both young and aged mice, although the response was blunted in aged animals when compared to young counterparts (Figure 2, unpublished results).

Intrigued by the dramatic upregulation of Klotho in response to a single acute exercise bout, we next investigated whether similar responses could be observed in a human population. We utilized a convenience sample of banked serum from “young” (age 36.0 ± 7.0 *SD* years; *n* = 12 females) and “older” (age 68.3 ± 3.0 SD years; *n* = 7 females) sedentary (exercised ≤1×/week) individuals to evaluate the change in circulating Klotho levels before and after completion of an acute exercise bout. In addition, we investigated whether completion of an exercise training protocol may affect the Klotho response to an acute exercise bout. Young women were mildly obese (BMI 30–38 kg/m^2^); older women varied between a BMI of 22–34 kg/m^2^. Exclusionary criteria included diagnosis of type 2 diabetes, coronary heart disease, peripheral vascular disease, or clinically significant hyperlipidemia. Individuals with treated or untreated hypertension were also excluded. The protocol was approved by the University of Pittsburgh Institutional Review Board, and written informed consent was obtained from each subject prior to participation.

The acute exercise bout for the young group consisted of one hour of treadmill walking at 55% VO_2_max, as determined using a standard incremental protocol (Swain and American College of Sports Medicine, 2014). Subjects were given a standard meal consisting of 10 kcal/kg of 50% carbohydrate- 30% fat-20% protein and then fasted overnight until the completion of the exercise bout (~10:00 am). Additionally, they were instructed to avoid strenuous physical activity for two days before the test. The young group then completed a 16-week progressive exercise training protocol consisting of four to six exercise sessions weekly, which primarily included cycling on a stationary bicycle, rowing or walking/jogging. At least one exercise session per week was supervised for each participant to assure that the target exercise intensity and duration was achieved. Following the 16-week training protocol, the young group completed the acute exercise bout again. The study protocol for the “older” group was essentially the same with the exception that the acute exercise bout was conducted on a cycle ergometer at 45% of VO_2_max and the exercise training protocol was for 12 weeks, and not 16 weeks as was performed for the young group. The fact that serum was collected from a banked convenience sample precluded matching training protocols exactly across age groups. For both young and older groups, blood was drawn a total of 4 times: before and after acute exercise both at baseline (ie. pre-training) and again post-training. Circulating Klotho levels were measured using a human soluble α-Klotho enzyme-linked immunosorbent assay Kit (Immuno-Biological Laboratories Co., Ltd., Takasaki, Japan).

**Figure 3:**
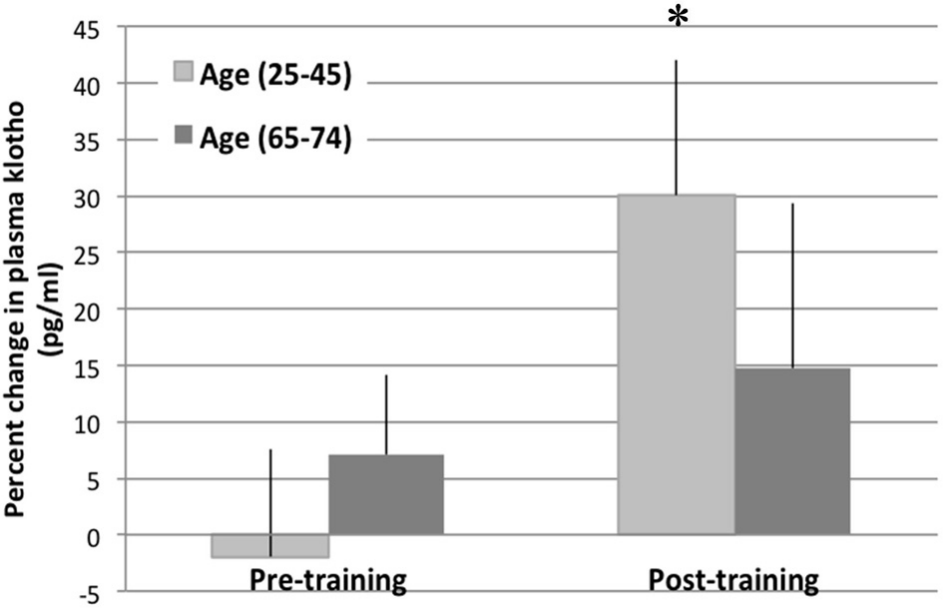
**Plasma Klotho levels following an acute bout of exercise in young and older individuals.** The change in plasma Klotho levels are depicted at baseline and immediately following an acute exercise bout (pre- and post-training). There was no change in circulating Klotho levels pre-training. However, post-training, there was a significant increase in circulating Klotho levels in the young (age 25–45 years) cohort after an acute bout of exercise. This response was blunted in the older cohort (age 65–74). ^*^denotes significantly different than pre-training values (within age group).

Prior to training, we observed no significant changes in the circulating levels of Klotho in response to an acute exercise bout [average change in circulating Klotho (pg/ml) pre-to-post acute exercise in young: −1.9% ± 9.53 *SE* and older: 7.06% ± 2.68 *SE*; Figure 3, unpublished results]. However, completion of a 16-week training program resulted in a significant increase in circulating Klotho levels in response to an acute exercise bout in young individuals [change in circulating Klotho (pg/ml): 30.08 ± 11.94%; *p* < 0.05, Figure 3]. Following training, older individuals also demonstrated an increase in circulating Klotho in response to acute exercise, although the effect of training was attenuated when compared to young counterparts [change in circulating Klotho (pg/ml): 15.25% ± 6.56 *SE*; *p* = 0.07 Figure 3, unpublished results].

Taken together, these findings in murine and human models suggest that exercise is a potent stimulus to increase plasma Klotho levels, but that the response may be dependent on physical fitness level as well as age. While this response to an acute exercise bout and training appears, from the current results, to be attenuated in older individuals, it should be noted that the training protocol for the aged individuals was slightly less intense and of a shorter duration than that of the young individuals. These differences in training intensity may confound the age-related differences in the response of Klotho to acute exercise and further clinical studies are warranted. However, in our murine studies, where the exercise intensity was matched across age groups, a similar age-related decline in the Klotho response following an acute exercise bout was also observed. Another intriguing observation came from the fact that individuals included in the pilot clinical study were overweight-obese. Interestingly, when the young participants were stratified according to BMI (<30 or >30 kg/m^2^) individuals with a lower BMI demonstrated significantly higher circulating Klotho levels after acute exercise (Average change = 10 ± 2.7% *SE*; *p* < 0.05), whereas those individuals in the higher BMI group demonstrated no change in circulating Klotho levels following acute exercise at baseline (−3.2% ± 7.5 *SE*; *p* = 0.40). However, following completion of a training program, individuals in the higher BMI group trended toward a significant increase in Klotho levels following an acute exercise bout (~30% increase; *p* = 0.06). These findings further support the hypothesis that the effect of an acute exercise bout on Klotho expression may be dependent on fitness levels. Future studies should investigate the effect of exercise intensity or duration on circulating Klotho levels.

Our findings in humans and mice that demonstrate an up-regulation of Klotho expression in response to acute activity demands are consistent with previous reports suggesting that Klotho plays an important role in energy metabolism. Recent studies have focused on the relationship between Klotho and peroxisome proliferator-activated receptor (PPAR) family members, which have been shown to act as lipid sensors to regulate energy metabolism. Specifically, PPAR-ϒ induces expression of the β-Klotho homolog (Zhang et al., 2008) and, conversely, β-Klotho upregulates PPAR-ϒ synthesis (Chihara et al., 2006), implicating the Klotho family of proteins as playing a role in lipid metabolism. Within the context of lipid oxidation, elderly individuals oxidize less fat during exercise when compared to young counterparts (Sial et al., 1996). However, training may be effective in reversing the age-related alterations in exercise response, and it is well established that, in both young and aged individuals, intramuscular fat oxidation during exercise is increased dramatically after training, an effect primarily attributed to an increased muscle respiratory capacity (Coggan et al., 1992; Proctor et al., 1995). One explanation for this benefit may come from the fact that training results in increased mitochondrial content, which thereby increases muscle respiratory capacity and promotes the utilization of fat over that of carbohydrate. Although excessive lipid accumulation is clearly detrimental to organismal health, maintenance of an adequate amount of lipid is clearly critical for maintaining physiological energy balance (Razzaque, 2012), and it is possible that the role of Klotho in maintaining this balance is mediated, at least in part, by skeletal muscle contractile activity. It would be interesting to investigate whether exercise-induced increases in Klotho may be a response to increased intramuscular fat oxidation as a feedback mechanism to maintain tissue lipid supplies under conditions of increased utilization. However, it should be noted that the role of Klotho in lipid metabolism has been linked to the β-Klotho homolog (reviewed in Kurosu and Kuro, 2009), whereas we specifically measured circulating α-Klotho levels in the current pilot studies. Further studies are needed to investigate how our observed exercise-induced changes in α-Klotho may be concomitant with alterations in β-Klotho. Alternatively, it is possible that increased skeletal muscle contractile activity induces a shift in calcium metabolism through Klotho-mediated FGF signaling, and that alterations in lipid metabolism may be a secondary effect of altered calcium homeostasis and mitochondrial biogenesis after exercise. Although further studies are needed to confirm the similarities between murine and clinical responses, the parallels observed in these preliminary studies suggest that pre-clinical investigations may serve as a good model for future mechanistic studies designed to interrogate the effect of physical fitness and skeletal muscle activity on the modulation of this potent longevity protein.

## Regulation of klotho in response to skeletal muscle injury

In addition to sarcopenia, increasing age typically results in a decreased overall skeletal muscle regeneration in response to damage (Jarvinen et al., 1983; Carlson and Faulkner, 1989; Brooks and Faulkner, 1990). Following injury, aged skeletal muscle demonstrates a shift from functional myofiber repair, as is typically seen in young individuals, to a “quick-fix” default toward fibrosis formation (Brack et al., 2007). This impaired response initiates a devastating cascade of muscle atrophy and weakness (Carlson and Faulkner, 1989), increased susceptibility to recurrent muscle injury (Croisier, 2004), and a prolonged recovery (McBride et al., 1995).

In young, healthy skeletal muscle, MuSCs are readily activated from a quiescent state in order to repair damaged myofibers (Mauro, 1961). While MuSC activation in young skeletal muscle often restores the original architecture and function of the damaged fibers, there is an age-related decrease in MuSC responses, as evidenced by MuSC differentiation toward a fibrogenic lineage (Brack et al., 2007), increased apoptosis (Ryall et al., 2008) and a decreased proliferation (Conboy et al., 2003). Declines in the activation of myogenic molecular pathways, including phosphatidylinositol 3-kinase (PI3K/Akt) signaling pathway, which directs cellular apoptosis, as well as Notch signaling, indispensable for MuSC proliferation immediately following injury (Conboy et al., 2003), have been implicated as culprits in age-related MuSC dysfunction. The mitogen activated protein kinase (MAPK) pathway, a positive regulator of Notch, is similarly age-responsive (Carlson et al., 2009a). Finally, it appears that decreased signaling for myogenesis is concomitant with increased activation of fibrogenic pathways owing to an age-related increased activation of the canonical Wnt signaling pathway, which contributes to a myogenic-to-fibrogenic conversion of MuSCs (Brack et al., 2007).

Fortunately, age-related changes in skeletal muscle regenerative potential are reversible, and several murine experiments have shown that rejuvenation of the systemic skeletal muscle microenvironment largely restores healing potential in aged mice (Carlson and Faulkner, 1989; Conboy et al., 2005; Brack et al., 2007). *In vivo*, heterochronic muscle transplantation experiments (Carlson and Faulkner, 1989) and parabiotic pairings, in which young and aged animals are surgically joined such that they share a common blood circulation, significantly enhances myofiber regeneration and decreases fibrosis formation of aged muscle following injury (Conboy et al., 2005; Brack et al., 2008). This enhanced tissue healing is concomitant with an inhibition of the Wnt signaling pathway and a decreased fibrogenic conversion of aged MuSCs (Brack et al., 2007). Importantly, it has been shown that the improved muscle healing of aged parabiotic partners is not the result of a physical contribution of the young cells within the circulation (Conboy et al., 2005). Taken together, these studies suggest that systemic niche factors play a critical role in dictating skeletal muscle regenerative potential, perhaps even more so than the intrinsic characteristics of the stem cells themselves. It has been hypothesized that the introduction of youthful factors into the circulation of aged partners inhibits, or functionally neutralizes, deleterious factors typically found in old animals (Carlson et al., 2009b).

There is precedence to suggest a relationship between Klotho expression and tissue regenerative capacity. In the skin, stomach, small intestine and kidney, the impaired tissue regenerative response of *kl* mice has been associated with a decreased stem cell frequency (Liu et al., 2007; Izbeki et al., 2010) and proliferation (Liu et al., 2007), impaired angiogenesis (Fukino et al., 2002), and decreased cellular resistance to stress (Yamamoto et al., 2005; Izbeki et al., 2010). In addition, the subcutaneous transplantation of bone marrow mesenchymal stem cells results in an attenuation of age-related degenerative processes and a concomitant lifespan extension of recipient mice, effects that were associated with an increased Klotho expression (Yamaza et al., 2009). Liu et al demonstrated that Klotho enhances stem cell regenerative potential and promotes tissue-healing through an inhibition of Wnt signaling activation (Liu et al., 2007). These latter findings were confirmed in recent studies demonstrating that, within the kidney, Klotho is able to directly bind to Wnt ligands extracellularly (Zhou et al., 2013). In the case of renal fibrosis, decreased Klotho expression was associated with an increased Wnt signaling activation and subsequent activation of fibrogenic signaling pathways (Zhou et al., 2013). Whether age-related declines in Klotho expression may contribute to the increased skeletal muscle Wnt signaling in aged animals, and thereby increased fibrosis deposition after injury, has not, to the best of our knowledge, been previously investigated.

Another potential mechanism by which Klotho may play be implicated as a potential regulator of skeletal muscle regeneration is through inhibition of Transforming Growth Factor-beta1 (TGF-β1) signaling. TGF-β1 is regarded as “master switch” for promoting mesenchymal transition toward a fibroblastic lineage in several tissues, including the kidney and lung (Willis and Borok, 2007; Doi et al., 2011). TGF-β1 ligands may be transported through the blood and bind to their specific receptors to initiate TGF-β-pSMAD signaling. With aging, circulating TGF-β1 expression in both mice and humans is increased, as is expression of the TGF-β1 receptors (Carlson et al., 2009b). It has been suggested that this may play a role in the increased fibrosis formation after injury of aged skeletal muscle (Li et al., 2004; Carlson et al., 2009b). Of note, physiological levels of TGF-β1 in young mice were predicted, based on *in vitro* investigations, to be inhibitory of MuSC myogenesis, suggesting that young sera may contain either a natural decoy of TGF-β1or a competitor to TGF-β1 signaling that inhibits the fibrotic cascade, and that the level of this decoy is decreased with increased age (Carlson et al., 2009b). Could Klotho act as such a decoy?

In a recent study by Doi et al., it was shown in the kidney that Klotho is indeed capable of interfering with TGF-β1signaling, but not TGF-β1 expression, in order to decrease myofibroblast infiltration and decrease fibrosis formation (Doi et al., 2011). Specifically, Klotho was shown to bind to the TGF-β1 receptor to inhibit TGF-β1 binding. Whereas Klotho depletion resulted in increased renal fibrosis, Klotho replacement therapy significantly alleviated the pathology, suggesting that decreased Klotho levels may contribute to the pathogenesis of renal fibrosis (Doi et al., 2011). Accordingly, systemic administration of a TGF-β1 receptor 1 (R1) kinase inhibitor effectively enhanced myofiber regeneration after injury, whereas application of a TGF-β1 neutralizing antibody had no effect (Carlson et al., 2009b). These findings support the above stated hypothesis that a natural decoy of TGF-β1 signaling may minimize activation of fibrogenic pathways in young animals following skeletal muscle injury, but that this suppression is lost in aged animals. Given the interactions between Klotho expression and TGF-β1 signaling, it is possible that age-related declines in circulating Klotho may result in a decreased opposition of TGF-β1 signaling, ultimately promoting fibrosis formation and impairing myofiber regeneration after injury. Moreover, it has been suggested in the kidney that Klotho effectively inhibits TGF-β1 activation of β-catenin, a downstream target of Wnt signaling, in tubular epithelial cells (Zhou et al., 2013). Future studies should explore the ability of Klotho to modulate Wnt signaling, potentially via inhibition of TGF-β1, in aged skeletal muscle models.

**Figure 4:**
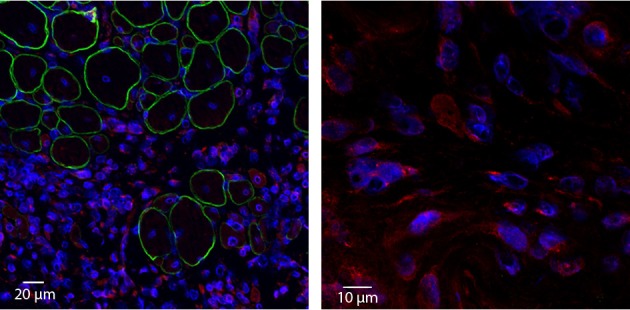
**Acute injury increases local Klotho expression within skeletal muscle.** Young (3–4 months old) wild type animals were exposed to a cardiotoxin injury to the tibialis anterior muscle. Two weeks after injury, TAs were harvested, cryosectioned and incubated with anti-Klotho (red), anti-Dystrophin (green) and nuclear (blue) antibodies (**left image**; 20× magnification). Corresponding high magnification image (**right image**; 100× magnification). Note that Klotho is undetectable in areas of more mature, regenerating myofibers. In contrast, strong expression of Klotho is observed in the area of cellular infiltrate.

Our preliminary studies support a potential contribution of Klotho to the skeletal muscle regenerative response. Two weeks following administration of an acute muscle injury to the tibialis anterior muscle of young wild type mice, we observed a dramatic increase in local Klotho expression (Figure 4, unpublished results). Importantly, Klotho was only expressed in the regions of cellular infiltrate and nascent myotubes, and not in the more mature regenerating myofibers (Figure 4). An important question is whether aged muscle displays an impaired Klotho response to injury as compared to young counterparts, and whether this decline contributes to the decreased regenerative capacity characterizing aged skeletal muscle. Future investigations should perform quantitative analysis of the response of Klotho to an acute injury event.

## Conclusions

History is replete with evidence demonstrating man's quest for a universal panacea to reverse the effects of aging in order to maintain and/or restore youthfulness. While a single “fountain of youth” remedy that counteracts all of the effects of time on organismal functioning is unlikely, an improved mechanistic understanding of tissue responses to the aging process and modifiable factors that may promote rejuvenation of these responses is desirable. Among such modifiable factors, physical activity has long been acknowledged for its anti-aging effects and the impact of skeletal muscle health on physical functioning and longevity is undeniable.

Indeed, systemic influences of skeletal muscle have been a growing topic of investigation and increased attention has been paid in recent years to the capacity of skeletal muscle to function as an endocrine organ capable of communicating with and dictating the behavior of distant organs. Modulation of Klotho expression through skeletal muscle contraction represents an intriguing relationship that may help explain the anti-aging effects of physical activity, and, as highlighted in this review, there is emerging evidence to suggest that such a relationship exists. Still unknown is whether skeletal muscle contractile activity itself results in the local production and secretion of Klotho into the bloodstream, or whether some myokine (a cytokine of skeletal muscle origin) is responsible for inducing Klotho expression in the kidney or brain, for example. This would be an interesting topic for future investigations. An improved mechanistic understanding of the potential role of skeletal muscle as a regulator of Klotho expression is important, as it may lead to the development of targeted and specific rehabilitation programs designed to counteract the effect of aging on organismal health.

### Conflict of interest statement

The authors declare that the research was conducted in the absence of any commercial or financial relationships that could be construed as a potential conflict of interest.
